# Articulatory movements modulate auditory responses to speech

**DOI:** 10.1016/j.neuroimage.2012.08.020

**Published:** 2013-06

**Authors:** Z.K. Agnew, C. McGettigan, B. Banks, S.K. Scott

**Affiliations:** Institute for Cognitive Neuroscience, University College London, 17 Queen Square, London WC1N 3AR, UK

**Keywords:** MEG, magnetoencephalography, PET, positron emission tomography, HRF, hemodynamic response function, IFG, inferior frontal gyrus, STG, superior temporal gyrus, pSTG, posterior superior temporal gyrus, pSTS, posterior superior temporal sulcus, mSTG, middle superior temporal gyrus, STS, superior temporal sulcus, BOLD, blood-oxygen level-dependant, FWHM, full-width half-maximum, MNI, Montreal Neurological Institute, FWE, family wise error, IPL, inferior parietal lobe, Speech, Motor, Auditory, Feedback, Agency

## Abstract

Production of actions is highly dependent on concurrent sensory information. In speech production, for example, movement of the articulators is guided by both auditory and somatosensory input. It has been demonstrated in non-human primates that self-produced vocalizations and those of others are differentially processed in the temporal cortex. The aim of the current study was to investigate how auditory and motor responses differ for self-produced and externally produced speech. Using functional neuroimaging, subjects were asked to produce sentences aloud, to silently mouth while listening to a different speaker producing the same sentence, to passively listen to sentences being read aloud, or to read sentences silently.

We show that that separate regions of the superior temporal cortex display distinct response profiles to speaking aloud, mouthing while listening, and passive listening. Responses in anterior superior temporal cortices in both hemispheres are greater for passive listening compared with both mouthing while listening, and speaking aloud. This is the first demonstration that articulation, whether or not it has auditory consequences, modulates responses of the dorsolateral temporal cortex. In contrast posterior regions of the superior temporal cortex are recruited during both articulation conditions. In dorsal regions of the posterior superior temporal gyrus, responses to mouthing and reading aloud were equivalent, and in more ventral posterior superior temporal sulcus, responses were greater for reading aloud compared with mouthing while listening. These data demonstrate an anterior–posterior division of superior temporal regions where anterior fields are suppressed during motor output, potentially for the purpose of enhanced detection of the speech of others. We suggest posterior fields are engaged in auditory processing for the guidance of articulation by auditory information.

## Highlights

► Using neuroimaging, we compare responses to normal speech production and perception. ► Sentences were voiced normally or mouthed silently while concurrently listening. ► Distinct regions of temporal cortex respond to auditory input from different sources. ► Inferior parietal regions are more sensitive to non-self generated auditory input. ► Silent articulation modifies auditory responses in superior temporal gyrus.

## Introduction

### Vocalization relies on auditory and somatosensory information

Many complex movements of the body are heavily dependent on sensory guidance, and different actions rely on different modalities to different extents. Movements of the upper limbs are highly dependent on visual information (and proprioception) for example (Jackson and Husain, 1996, 1997), whereas movements of the face, mouth and articulators rely to a large extent on auditory and somatosensory information (Rauschecker and Scott, 2009). In the case of vocalization, both somatosensory and auditory information play an important role in guiding movement (Tremblay et al., 2004). Interference with somatosensation, through use of anesthetics or mechanical perturbation, results in changes to speech production that can to some extent be compensated for by the speaker (Burke, 1975; Nasir and Ostry, 2009). This indicates that, at least in abnormal circumstances, somatosensation can modulate articulation. Similarly, during vocalizations, the perturbation of auditory feedback results in altered production of vocalizations, an effect that is not limited to humans (Brainard and Doupe, 2000; Leonardo and Konishi, 1999). In humans, changes to the apparent pitch, spectrum or timing of the produced speech can result in many different speech errors, yet frequently subjects are able to compensate for these sensory changes (Tourville et al., 2008). However what remains unclear is how auditory input is processed when afferent auditory signals are a consequence of motor output (normal speech) compared with when the auditory input is not a product of a self-generated articulation. The aim of this study is to address this issue directly by comparing the neural correlates of articulation, with and without self-produced speech.

### Not all parts of the auditory pathways respond in the same way during self-made and externally produced vocalizations

Despite the importance of auditory information on maintaining motor output (e.g. via incoming sensory mechanisms), activity in many nodes of the ascending auditory pathway is attenuated during self-made vocalizations. In humans and other animals, the amplifying mechanisms of the middle ear are dampened during vocalization in order to reduce the auditory consequences of self-made sounds (Carmel and Starr, 1963; Salomon and Starr, 1963; Suga and Jen, 1975). A number of studies have demonstrated that cortical responses to self-made vocalizations may also be attenuated. In non-human primates, single cell recordings have demonstrated that self-produced vocalizations and those of others are processed differently in the same cell populations. Eliades and Wang (2003) report that of the cells investigated in auditory cortex, the majority of cells showed a suppression of activity (vocalization-induced suppression), whereas a small number of cells increased their activity during self-produced vocalization. The two populations of cells responded differently to additional auditory input during vocalization and it issuggested that the suppression is driven by vocal production. In humans a similar dampening of the auditory response to self-vocalizations has been demonstrated with MEG (Curio et al., 2000; Gunji et al., 2001), PET (Paus et al., 1996; Wise et al., 1999) and intracranial recordings (Creutzfeldt et al., 1989; Flinker et al., 2010; Greenlee et al., 2011).

A dissociation between how self- and externally generated actions are encoded in the visual domain has recently been demonstrated. Kontaris et al. (2009) showed that different parts of networks for processing observed biological motion respond differently when the observed action matches the one produced by the observer. They demonstrate that an early visual processing area (the fusiform face area), does not discriminate between observed actions of self and other, but downstream posterior superior temporal sulcus (pSTS) responded more strongly to actions produced by others.

The aim of the current study is to investigate how sensory responses differ for self-produced and externally produced speech and to investigate whether the same networks are engaged for auditory perception and for control of speech production. BOLD responses were measured during reading aloud, silently mouthing of speech while listening to another speaker, or passive listening to the speech. In this way we were expressly able to address regions suppressed during motor output and the differential processing of self- and externally generated speech stimuli.

## Methods

### Design and materials

The conditions in the experiment were:

*Reading aloud* (ReadAloud_(ownvoice)_)
*Silent mouthing while listening* (MouthSilently_(othervoice)_).
*Passive listening* (ReadSilently_(othervoice)_)
*Covert reading of text* (ReadSilently_(novoice)_)

In order to construct all the required conditions, we required auditory recordings from a corpus and visually presented sentences from the same corpus for motor output conditions. All stimuli were generated from the IEEE corpus (IEEE, Institute of Electrical and Electronic Engineers, 1969), for example ‘The birch canoe slid on the smooth planks'. In order to make the auditory stimuli for the silent articulation with listening condition, sentences were produced by a variety of speakers as part of a behavioral study. All speech stimuli were produced by native British speakers which comprised both male and female speakers with a range of regional accents. Listening to recordings of one's own speech is not necessarily an appropriate control for auditory input during speech production for two reasons. First, we primarily hear our own voices through bone conduction which emphasizes lower frequencies better than air conduction. Second, as we hear our own voices from two sources, through bone conduction and through the ear canal, it is very difficult to recreate the spatial characteristics, as well as the spectral characteristics of one's own voice realistically. We therefore used speech recorded from a range of British speakers such that everybody heard the same male and female speakers. Text was presented using Psychophysics toolbox running on Matlab 7.4 (Mathworks Inc., Sherborn, MA). Speech stimuli were recorded using a solid state recorder (Edirol, R-09HR) at 24 bits, 96 kHz, and saved as wav files. The sound files were normalized using the peak amplitude in Praat (Boersma and Weenink, 2010).

### Subjects

Twenty healthy right-handed subjects (mean age 26 years ± 5, 11 female) participated in the present study. All were native English speakers and we excluded any subjects who had any history of speech or hearing deficits. All gave informed consent according to the guidelines approved by the UCL Ethics Committee who provided local ethics approval for this study.

### fMRI

A 1.5 T Siemens Avanto system (Siemens AG, Erlangen, Germany) in combination with a 12 channel head coil was used to acquire 180 T_2_*-weighted whole brain echo-planar images (EPI) data (3 × 3 × 3 mm^3^ in plane resolution, TR = 10s, TA = 3 s, TE = 50 ms, flip = 90°, 35 slices) using blood-oxygen level-dependent (BOLD) contrast. A sparse scanning protocol was employed in order to administer the auditory stimuli in the absence of scanner noise and minimize artifacts related to head motion during speech production. The repetition time (TR) for each trial was 10 s, during which time subjects were asked to either speak, mouth, listen to or silently read a sentence within a 4 second time window. Following a silent delay of 2 s, a single volume (3 s) was collected for each trial before a further 1 second silent period. Instructions for the following trial were presented during acquisition. The first two functional volumes were discarded in order to remove the effect of T_1_ equilibration. High resolution T_1_ anatomical volume images (HIRes MP-RAGE160 sagittal slices, voxel size 1 mm^3^) were also acquired for each subject. During the experiment subjects lay supine in the scanner in the dark and were asked to pay attention to instructions presented on a screen.

Sounds and instructions were presented using Matlab 9b (Mathworks, Sherborn, MA) with the Psychophysics Toolbox extension (Brainard, 1997), via a Denon amplifier (Denon UK, Belfast, UK) and electrodynamic headphones fitted with an optical microphone were worn by the participant (MR Confon GmbH, Magdeburg, Germany). Instructions were projected from a specially-configured video projector (Eiki International, Inc., Rancho Santa Margarita, CA) onto a custom-built front screen, which the participant viewed via a mirror placed on the head coil. Speech output was recorded using Audacity (http://audacity.sourceforge.net/).

Each trial comprised a visually presented instruction followed by the presentation of a single sentence from the IEEE list. The instructions were either, ‘Listen’, ‘Read Aloud’, ‘Mouth Along’ or ‘Read Silently’. In all four conditions, the instruction was followed by the visual presentation of a sentence so that all conditions were matched for reading. Subjects were told that following a ‘Listen’ instruction, a sentence would appear on the screen and then that same sentence would be played to them over headphones. They were told to read the sentence on the screen and passively listen to the audio recording (ReadSilently_(othervoice)_). Following the ‘Read Aloud’ instruction, subjects were told to read aloud the subsequent sentence as normally as possible (ReadAloud_(ownvoice)_). Sparse scanning enabled subjects to hear their own voices both via normal audition and through bone conduction as the speech production part of each trial was performed in the absence of scanner noise. On seeing a ‘Mouth Along’ instruction subjects had to silently articulate the sentence on the screen, while the same sentence was played over the headphones by a different speaker (MouthSilently_(othervoice)_). Subjects were trained until they could perform this task without issue before entering the scanner. For the high-level baseline condition, subjects saw the instruction ‘Read Silently’, upon which subjects were told to read the sentence silently (ReadSilently_(novoice)_), in order to control for semantic and linguistic processing associated with silent reading of written words.

Participants were trained on these instructions outside of the scanner until they were familiar with the task. There were 30 examples of each condition played in a randomized order. This lasted approximately 35 min which was carried out in a single fMRI session.

### Pre-processing and analyses

Functional data were analyzed using SPM8 (Wellcome Department of Imaging Neuroscience, London, UK) running on Matlab 7.4 (Mathworks Inc., Sherborn, MA). All functional images were realigned to the first volume by six-parameter rigid-body spatial transformation. Functional and structural (T_1_-weighted) images were then normalized into standard space using the Montreal Neurological Institute (MNI) template. Functional images were then coregistered to the T_1_ structural image and smoothed using a Gaussian kernel of full-width half-maximum (FWHM) of 8 mm. The data were high-pass filtered at 128 Hz. First level analysis was carried out using motion parameters as regressors of no interest at the single-subject level whereby each trial was modeled using a canonical hemodynamic response function (HRF) beginning at the onset of each trial (i.e. the onset of speech production, mouthing or the onset of the auditory stimulus). Individual contrasts were carried out to investigate the BOLD response to each condition minus the silent reading condition, and relative to each other. These contrast images were taken up to a second level random effects model. At the group level, contrasts (each condition compared the baseline of silent reading, ReadSilently_(novoice)_), were thresholded using a family wise error at p < 0.05. All further comparisons were thresholded at p < 0.005 and in all cases voxelwise thresholding was carried out at 20 voxels to limit potential type II errors. The reason for using a more conservative correction method for the basic contrasts (compared with passive reading) is that speech production, silent articulation and passive listening activate extremely large amounts of the cortical surface. Comparisons between the conditions are more subtle and therefore require a more liberal threshold and corrective method. A conjunction null (Nichols et al., 2005) identifies voxels that are significantly active in more than one contrast. This is done by taking the intersection mask of two thresholded images so that it is possible to look at voxels that are significantly active in the contrast (A > B) and also in the contrast (C > D). These were carried out using a masking threshold of p < 0.001. Significant BOLD effects were rendered on a normalized template. Region of interest analyses were carried out to investigate mean effect sizes in specific regions across all experimental conditions against baseline, using the MarsBar toolbox that is available for use within SPM8 (Brett et al., 2002).

## Results

**Fig. 1:**
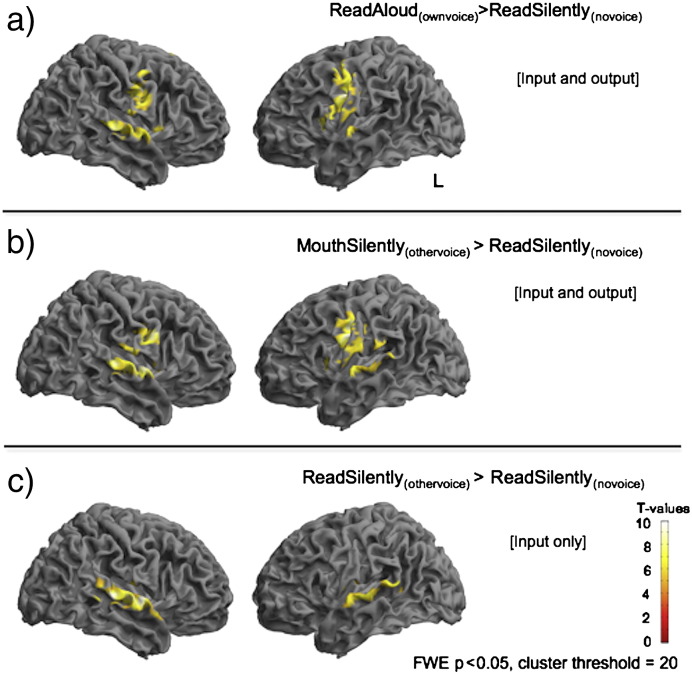
Speech production, silent articulation and passively listening compared with silent reading. When compared with covert reading (ReadSilently_(novoice)_), speech production [ReadAloud_(ownvoice)_] is associated with activity in bilateral middle and posterior superior temporal gyri with more distributed activity in the right, and large clusters comprising peaks in ventral somatosensory and premotor and primary motor cortices (1a). Silent articulation with passive listening [MouthSilently_(othervoice)_] is a condition where the motor output and auditory input is comparable to normal speech production but the auditory input and motor output are incongruent, i.e. the auditory input is not a direct result of the motor output. Compared with covert reading, this condition was associated with a very similar pattern of activity to normal speech production, including middle STG and ventral somatosensory and motor areas. Visual inspection indicates that activity in the right hemisphere extended posteriorly compared with the [ReadAloud_(ownvoice)_] condition seen in the top panel. Finally passive listening [ReadSilently_(othervoice)_] was associated with significant BOLD activity in dorsolateral temporal cortex in both hemispheres but again, with more distributed activity in the left. These contrasts are all corrected using a family wise error correction at a threshold of p < 0.05, with a 20 voxel cluster threshold. For full lists of significant peaks see Table 1.

Fig. 1 shows the comparison of each of the main experimental conditions with the baseline of covert reading (ReadSilently_(novoice)_). Both auditory-motor conditions (ReadAloud_(ownvoice)_ and MouthSilently_(othervoice)_) were associated with activity in ventral primary and premotor regions and superior temporal cortices (Figs. 1a and b), whereas the passive listening condition (ReadSilently_(othervoice)_) was associated with activity in superior temporal gyri only (Fig. 1c). These basic contrasts were thresholded at FWE p < 0.05, with a cluster extent of 20 voxels, in order to constrain widespread activity to key regions.

### Comparison of auditory and motor processing during active and passive states

**Fig. 2:**
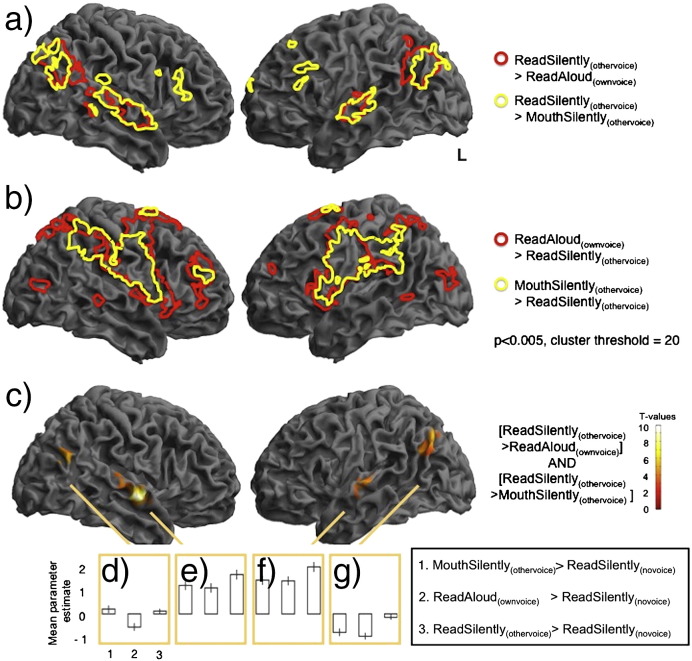
Passive listening was associated with increased activity in STG and IPL compared with both motor output conditions despite comparable auditory input. All three main conditions, ReadAloud_(ownvoice)_, MouthSilently_(othervoice)_ and ReadSilently_(othervoice)_ involved comparable auditory input. In order to look at how motor production modulates sensory processing, BOLD activity during passive listening was compared with both the auditory motor conditions: ReadSilently_(othervoice)_ compared with ReadAloud_(ownvoice)_ was associated with significant activity in middle superior temporal gyri and inferior parietal cortices in both hemispheres (Fig. 2a, red outline). ReadSilently_(othervoice)_ compared with MouthSilently_(othervoice)_ was associated with activity in the same regions but extended to inferior frontal gyrus and with a peak in left medial STG/ parietal operculum (Fig. 2a, yellow outline). The reverse contrasts revealed widespread activity in significant activity in bilateral ventral motor, premotor and somatosensory cortices, inferior parietal cortex, inferior frontal cortex and supplementary motor area (Fig. 2b, red and yellow outlines). In order to look directly at the overlap between activity greater for listening compared with the two auditory motor conditions seen in Fig. 3a, a null conjunction was performed of [ReadSilently_(othervoice)_ > ReadAloud_(ownvoice)_] and [ReadSilently_(othervoice)_ > MouthSilently_(othervoice)_] using a masking threshold p < 0.001. This revealed significant activity common to both comparisons in middle to posterior STG and inferior parietal cortices in both hemispheres (Fig. 2c). The parameter estimates for these four clusters were extracted and are plotted in the bottom three panels (Figs. 3d–g) for the contrasts [MouthSilently_(othervoice)_ > ReadSilently_(novoice)_], [ReadAloud_(ownvoice)_ > ReadSilently_(novoice)_], [ReadSilently_(othervoice)_ > ReadSilently_(novoice)_], respectively. These plots demonstrate that inferior parietal regions that respond preferentially to passive listening are suppressed for normal speech (Graphs d and g), but regions in the superior temporal cortex that preferentially respond during passive listening are also active during the two production conditions, but to a lesser degree (Graphs e and f). Despite comparable auditory input, there is more activity in these four regions during passive listening than articulation. This indicates that something about producing a motor articulatory output, whether it be silent movement or not, is modulating activity in these regions (all maps are thresholded at p < 0.005, cluster threshold 20).

In order to look at how auditory processing compares during active and passive motor states, we compared passive listening, (ReadSilently_(othervoice)_), with both ReadAloud_(ownvoice)_ and MouthSilently_(othervoice)_ conditions. We report significantly increased activity in middle superior temporal gyri in both hemispheres, with greater extent on the right, and bilateral inferior parietal cortices during ReadSilently_(othervoice)_ compared with either ReadAloud_(ownvoice)_ (Fig. 2a, red line) or MouthSilently_(othervoice)_ conditions (Fig. 2a, yellow line). The reverse contrasts ([ReadAloud_(ownvoice)_ > ReadSilently_(othervoice)_]: Fig. 2b, red line, [MouthSilently_(othervoice)_ > ReadSilently_(othervoice)_], Fig. 2b, yellow line), revealed significant activity in bilateral ventral motor, premotor and somatosensory cortices, inferior parietal cortex, inferior frontal cortex and supplementary motor area. In the case of [ReadAloud_(ownvoice)_ > ReadSilently_(othervoice)_] this activity extended to the lateral occipital cortex. The null conjunction (masking threshold p < 0.001) of [ReadSilently_(othervoice)_ > ReadAloud_(ownvoice)_] and [ReadSilently_(othervoice)_ > MouthSilently_(othervoice)_] revealed significant common activity in bilateral middle superior temporal cortices and inferior parietal cortex corresponding to the posterior end of the angular gyri (Fig. 2c). Mean parameter estimates were extracted from spherical regions of interest based around these four peaks. The results, plotted in Figs. 2d and g, reveal that despite very similar auditory input in all three conditions, the two inferior parietal regions that respond preferentially to listening are suppressed for normal speech. In contrast, temporal regions that are preferentially responsive during listening are also active during the two production conditions, just to a lesser extent (Fig 2, graphs e and f, for full lists of significant peaks see Table 1, supplementary information).

### Comparison of two auditory motor conditions

**Fig. 3:**
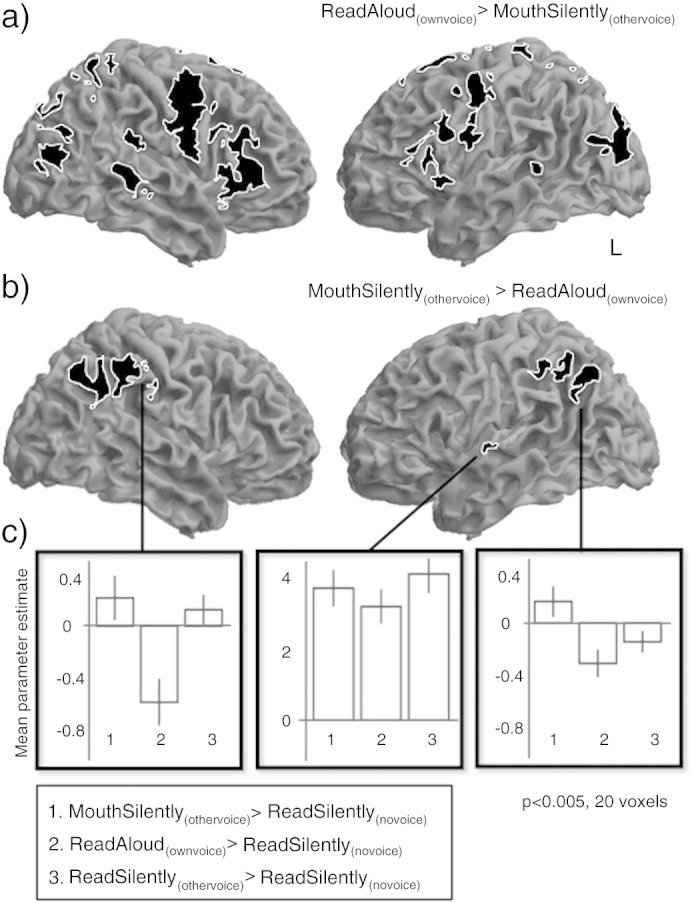
Comparison of auditory motor conditions differentiates between aspects of the auditory pathway that encode self-generated vocalizations. The congruent and incongruent speech conditions are formed of normal speech production [ReadAloud_(ownvoice)_], or silent articulation while listening to the same sentence, spoken by someone else played back at the same time [MouthSilently_(othervoice)_], respectively. A direct comparison of the two [ReadAloud_(ownvoice)_ > MouthSilently_(othervoice_] revealed significant activations of widespread motor cortices (premotor, inferior frontal, supplementary motor and anterior insula), superior temporal cortex and occipital cortex, (Fig. 3a). The opposite contrast, [MouthSilently_(othervoice_ > ReadAloud_(ownvoice)_], revealed significant activity in bilateral inferior parietal cortex, including both supramarginal and angular gyri (3b). Mean parameter estimates were extracted for the inferior parietal and temporal clusters and are shown in the bottom two panels (Fig. 3c), demonstrating that in both peaks, activity was not only much less for the [ReadAloud_(ownvoice)_ condition] than the listening [ReadSilently_(othervoice)_] condition but was also below baseline, indicating suppression of activity (all maps are thresholded at p < 0.005, cluster threshold 20).

A direct comparison of the auditory-motor conditions (ReadAloud_(ownvoice)_ and MouthSilently_(othervoice)_) revealed significant differences between the two patterns of activity, despite the similarities in the motor output and auditory input. In the comparison of [ReadAloud_(ownvoice)_ > MouthSilently_(othervoice)_], significant activity was seen in motor cortices (premotor, inferior frontal, supplementary motor and anterior insula), superior temporal and occipital cortex (Fig. 3a, Table 1). The opposite contrast [MouthSilently_(othervoice)_ > ReadAloud_(ownvoice)_] revealed significant activity in two large clusters spreading over bilateral inferior parietal cortices including primary somatosensory cortex, supramarginal and angular gyri, and a small cluster on the lateral surface of the left mid-anterior superior temporal gyrus (Fig. 3b). The three clusters generated by this contrast were used to create regions of interest from which mean parameter estimates were extracted and plotted in Fig. 3c. These plots demonstrate that in both parietal clusters, activity was not only much less for the ReadAloud_(ownvoice)_ condition than the ReadSilently_(othervoice)_ condition but was also below that of the ReadSilently_(novoice)_ baseline, indicating suppression of activity. Again, the profile of activity in superior temporal indicates a graded response to all three conditions, where responses were all above baseline, but greatest for listening and least for reading aloud.

### Separate regions within the dorsolateral temporal cortex respond during speech production, silent mouthing while listening, and passive listening

**Fig. 4:**
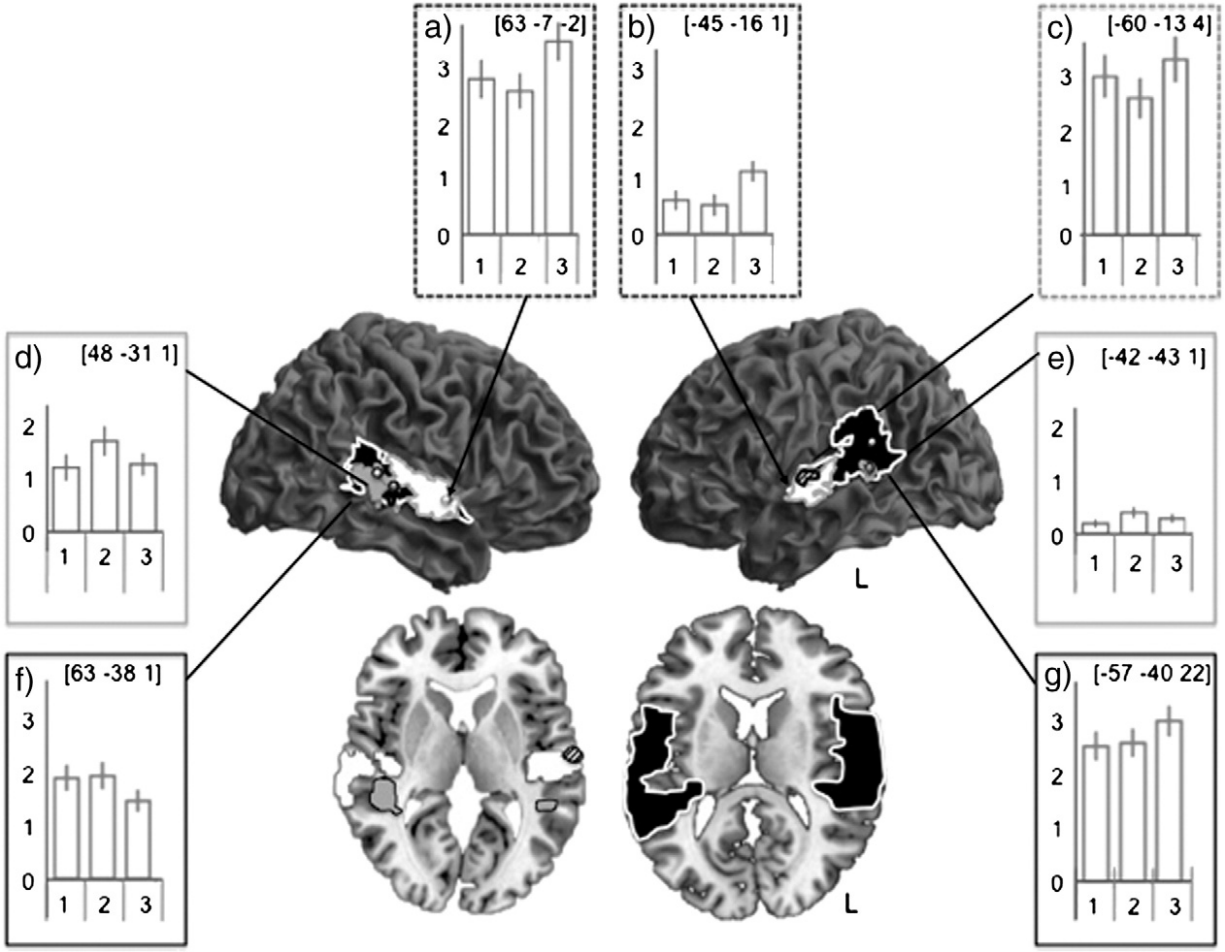
Separate clusters in superior temporal cortex respond during speech production and listening. All three main conditions were associated with widespread activity across the dorsolateral temporal cortices in both hemispheres. In order to look at how response profiles differed across conditions in the temporal cortex, Fig. 4 displays only peaks lying in the superior temporal cortex for all conditions, revealing an anterior–posterior distribution. Passive listening was associated with significant activity in the most anterior cluster. A null conjunction of [ReadSilently_(othervoice)_ > [ReadAloud_(ownvoice)_] and [ReadSilently_(othervoice)_ > [MouthSilently_(othervoice)_] is shown in yellow, (graphs 4a and 4b). A render of this cluster on an axial slice demonstrates that this anterior cluster extends from the lateral surface to the medial extent of the superior temporal gyrus. Lying within the more middle temporal aspects of the anterior cluster, on the left medial surface is a small peak (green) which is more active for [MouthSilently_(othervoice)_] compared with [ReadAloud_(ownvoice)_]; the plot of activity within this region (graph 4c) demonstrates that this is due to activity in this small cluster being less suppressed for the mouthing condition than for normal speech production. A more posterior and inferior region (pSTS, shown in red, graphs d and e) is more active for normal speech production compared with mouthing while listening [ReadAloud_(ownvoice)_] > [MouthSilently_(othervoice)_]. This pSTS cluster extends medially as can be seen on the axial slice. Finally at the posterior and superior extent of the superior temporal gyrus lies a cluster that is commonly active for both auditory motor conditions, shown in blue (null conjunction of [ReadAloud_(ownvoice)_] + [MouthSilently_(othervoice)_], p < 0.001, graphs 4f and g). Solid lines indicate the cluster and spheres indicate the peak of each cluster. Spherical regions of interest of 3 mm radius were extracted for each peak coordinate and mean parameter estimates were extracted. These are plotted for each peak in graphs a–g where the y axis represents the mean parameter estimate and the three bars represent the mean parameter estimates for 1: [MouthSilently_(othervoice) >_ ReadSilently_(novoice)_] 2: [ReadAloud_(ownvoice)_ > ReadSilently_(novoice)_] and 3: [ReadSilently_(othervoice)_ > ReadSilently_(novoice)_]. All clusters are thresholded at p < 0.005, using a cluster extent of 20 voxels.

Fig. 4 displays a summary of the clusters revealed in the above comparisons that are present in the superior temporal cortex. This approach reveals separate fields within the dorsolateral temporal cortex that are active for different auditory and auditory motor conditions. The most anterior fields are more active for passive listening than for either of the auditory motor conditions, despite the fact that auditory input in all three conditions is comparable (Figs. 4a and b, yellow, null conjunction of [ReadSilently_(othervoice)_ > [ReadAloud_(ownvoice)_] and [ReadSilently_(othervoice)_ > [MouthSilently_(othervoice)_]). This region encompasses middle-anterior STG in both hemispheres that extends from the lateral surface medially to encompass the supratemporal plane. Within this large cluster in the left hemisphere, there is a small cluster which is more active during reading aloud compared with silent mouthing (Fig. 4c, green, [ReadAloud_(ownvoice)_ > [MouthSilently_(othervoice)_]). The activity profile for this cluster demonstrates that the effect is driven by reduced suppression for silent articulation during listening, than for reading aloud with normal feedback. In contrast to the profiles in anterior STG, the comparison of reading aloud and silently mouthing while listening (Figs. 4d and e, red, [ReadAloud_(ownvoice)_ > MouthSilently_(othervoice)_]) revealed significant activations at the posterior end of the superior temporal sulcus (pSTS) in both hemispheres, with a more distributed pattern of activity on the right. Finally we looked at regions that were commonly activated for both auditory-motor conditions by looking at the null conjunction of [ReadAloud_(ownvoice)_ + MouthSilently_(othervoice)_] (Figs. 4f and g, purple). This revealed a large cluster lying at the posterior end of the STG, extending ventrally to the region in the pSTS that distinguished between these two conditions.

## Discussion

The present study investigated how sensory cortical fields are modulated by articulation. There was considerable activation in motor and premotor cortex for speaking and mouthing (Fig. 2) but we focus our discussion on the responses in the temporal and parietal lobes as the study was designed to address specific aspects of the sensory consequences of speaking. First, we show vocalization-induced suppression of activity during speech production in superior temporal and inferior parietal cortex in both hemispheres and we report for the first time that silent articulation while listening is sufficient to modify responses in dorsolateral temporal cortex. Second, we demonstrate that within bilateral inferior parietal cortex and left superior temporal cortex, silent mouthing while listening is associated with increased activity relative to speech production. This is despite the comparable levels of motor output and auditory input across mouthing and speaking aloud. Finally, we report an anterior–posterior division of activity profiles within the dorsal temporal cortices. These results are discussed in detail below with reference to current models of speech production – the DIVA model (Tourville and Guenther, 2011) and Hierarchical State Feedback Control model (Hickok, 2012) – and other empirical work on auditory-motor interactions in speech production.

### Vocalization induced suppression during silent mouthing

It is well established that self-made vocalizations are accompanied by suppression of activity in superior temporal fields. This has been shown in non-human primates (Eliades and Wang, 2003, 2005, 2008) and in humans using a range of techniques (Curio et al., 2000; Flinker et al., 2010; Gunji et al., 2001; Paus et al., 1996). Here we confirm this effect using functional neuroimaging, by demonstrating that mid-anterior regions of bilateral STG are more active for passive listening than for speech production. We also demonstrate, for the first time, that silent mouthing of words while listening is sufficient to produce this suppression of activity in the same mid-anterior cortical fields. This result suggests that a motor act that does not have expected auditory consequences is sufficient to initiate suppression. A recent theoretical paper modeled this suppression of auditory areas during speech production, as a result of matching of expected and actual auditory and motor representations (driven by lemma activations) in the temporal cortex (Hickok, 2012). Our results are inconsistent with this model, as we show that silent articulation is sufficient to drive suppression in anterior superior temporal fields.

### IPL activity during in the articulatory conditions: ReadAloud_(ownvoice)_ compared with MouthSilently _(othervoice)_

We report significantly greater activity in inferior parietal cortex during silent mouthing and listening, compared with reading aloud. Moreover, activity in this region was below baseline for reading aloud, indicating a suppression of activity. Dhanjal et al. (2008) demonstrated suppression of activity, albeit in a slightly more anterior portion of the inferior parietal cortex, during propositional speech compared with silent movements of the jaw and tongue. The DIVA model of speech production proposes a modality specific error monitoring system whereby inferior parietal regions encode somatosensory differences (‘error’) between the predicted and actual somatosensory consequences of a vocalization, and STG encodes auditory ‘error’; Tourville et al. (2008) suggest that BA40 (supramarginal gyrus) is the orosensory area where somatosensory representations of speech are processed following projections containing motor efference copy from premotor cortex (BA6). In the current experiment, the MouthSilently_(othervoice)_ condition may have incurred such auditory errors but not somatosensory, yet we report activity in both IPL and STG in relation to this condition. Thus the modality specific nature of the DIVA model does not predict a difference between unperturbed speaking aloud and unperturbed mouthing, thus the anatomical predictions made by the model do not account for the present data.

A recent study that has specifically compared neural responses during speech production and silent movements of the tongue has reported suppression in an inferior parietal region that overlaps with regions reported here during reading aloud (Geranmayeh et al., 2012). This suggests that in the present study, activity in this region is not due to the unexpected auditory input in the mouthing condition. The authors attribute this response profile in the IPL, in part, to increased activity in the default mode network during silent movements of the tongue, but their use of independent component analysis also identified a contribution of the IPL to task related effects. Similarly it has been shown that the production of meaningless speech (repetitive syllable production) and writing (repetitive grapheme production) is associated with activity in bilateral IPL when compared with meaningful speech and writing (Brownsett and Wise, 2010).

The junction of temporal and parietal cortex is thought to be involved in representing sensory-motor properties of sounds (e.g. in order to mimic them), or as a representation of the human vocal tract (Hickok, 2009), and comprises part of an auditory ‘how’ pathway responsible for auditory motor transformations (Rauschecker and Scott, 2009) which extends to the inferior and posterior parietal cortex (Rauschecker, 2011). It has been suggested that the projection from caudo-lateral STG to inferior and posterior parietal areas subserves more than just linking sounds to their motor representations but also serves to match predicted sensory outcomes to afferent input (Rauschecker, 2011). The inferior parietal cortices have also been argued to contain an internal model for vocalization (Wolpert et al., 1995). Stimulation of supramarginal and angular gyri is known to elicit dysphasia (Van Buren et al., 1978) and recent work has identified a role for inferior parietal cortex in motor learning in speech (Shum et al., 2011). Shum and colleagues demonstrated that repetitive TMS to the left supramarginal gyrus disrupts the ability of subjects to adapt to enforced changes in sensory feedback but left normal speech production unaltered. These data led the authors to suggest that STG comprises an important part of auditory monitoring and unpredictable error detection during normal over-learnt motor actions such as normal speech, and that the supramarginal gyrus is of central importance in the presence of predictable error signals necessitating adjustment of internal models. Our data fit with this interpretation, and in fact suggest that a slightly more posterior region of the IPL is suppressed during normal speech production compared with a silent mouthing with concurrent listening, a condition in which an error signal is likely to occur.

### Modulation of activity in dorsolateral temporal cortex by articulation; anterior–posterior profiles of activity

We report three distinct response profiles, associated with speech production, listening and silent mouthing while listening, in superior temporal cortex (Fig. 4). Activity in bilateral anterior superior temporal regions was greater for passive listening, compared with normal vocalization or silent articulation with concurrent auditory input. This suggests, along with previous studies (Curio et al., 2000; Gunji et al., 2001; Wise et al., 1999; Creutzfeldt et al., 1989) that anterior temporal fields are suppressed during speech production and silent mouthing. Moving posteriorly, there is a small cluster in left middle STG which displays a significantly greater response to the silent mouthing while listening compared with reading aloud. Our data demonstrate that in the left STG region, there is *less* suppression during silent mouthing and listening, than for reading aloud. This is interesting in light of recent work demonstrating that self-generated vocalizations are perceived as quieter than externally or digitally produced sounds (Weiss et al., 2011). This might reflect some of the error monitoring processing predicted by the DIVA model, since in the mouthing condition there is an unexpected auditory input. Alternatively, this response might reflect obligatory perceptual processing of the speech produced by the other speaker since during mouthing, there was always another talker producing the same sentence.

In the posterior temporal lobes we find activity associated with speech production over and above silent articulation and listening in a set of regions comprising auditory and motor areas (premotor, inferior frontal, anterior insula and supplementary motor cortex). The posterior STS cluster lies in a region posterior, inferior and medial to regions that are suppressed during speech production. Previous studies have reported similar activations for speech production compared with silent movements of the articulators (Geranmayeh et al., 2012; Wise et al., 2001). Dorsally, there was extensive activation of bilateral posterior superior temporal gyri by speaking aloud and mouthing, relative to listening. Also forming part of the ‘how’ pathway (Rauschecker and Scott, 2009), these posterior auditory areas are known to respond to auditory spatial cues and vocal sounds and it has been suggested that the posteromedial supratemporal plane serves as an auditory motor interface (Hickok and Poeppel, 2007; Warren et al., 2005; Wise et al., 2001). More specifically, it is suggested that this region matches auditory input with auditory templates in order to constrain motor output. A more recent suggestion is that higher order levels of processing are responsible for predicting the auditory and somatosensory consequences of a planned action (Price et al., 2011). Price and colleagues show that silent articulation of speech sounds (i.e. movements normally associated with auditory consequences) compared with silent non-speech mouth movement (with no, or less auditory associations) activates left IFG and pSTG regions. This is interpreted as evidence that auditory and motor aspects of an internal model for speech production are encoded in the left IFG and that phonological processing that underlies prediction of auditory response occurs in the left pSTG. In contrast to this, Rauschecker (2011) suggests that the posterior superior temporal/parietal cortex represents the internal model for speech production. In light of this latter suggestion, we may be seeing differences in this region reflecting the different demands of speaking and mouthing. For example, it is well established that metabolic breathing is associated with widespread motoric activity (Simonyan et al., 2009) and that breathing differs greatly during speech production and silent articulation (Murphy et al., 1997).

### Implications for models of speech production

In terms of speech perception, it is well established that STG is an anatomically (Pandya and Sanides, 1973) and functionally (Rauschecker and Scott, 2009) heterogeneous region, with an anterior stream encoding identification of auditory input and a posterior stream linking auditory input to motor representations. In current models of speech production, no such distinction is made within the superior temporal cortex (Hickok, 2012; Tourville and Guenther, 2011). The present data indicate that there may also be an anterior–posterior distribution of functional roles within the temporal lobes whereby anterior regions are suppressed during speech production, possibly as a consequence of efference copy originating from vocal production centers (Eliades and Wang, 2003), and posterior regions are more responsive during production of sound that have auditory consequences. We suggest that posterior temporal fields may serve to provide some form of sensory guidance of motor output as these regions are consistently activated when sensory consequences are perturbed (Hashimoto and Sakai, 2003; Takaso et al., 2010; Watkins et al., 2005). Within these posterior regions we report two separate peaks, one lying dorsally, extending to the medial extent of the supratemporal plane and one lying inferiorly, in the STS but also extending medially. The former lies within a region that has been suggested to comprise auditory motor interface (Hickok and Poeppel, 2000; Warren et al., 2005; Wise et al., 2001) and in the present study responds during speech production and mouthing while listening. Conversely the more inferior pSTS region is more active for speech production than mouthing, which has been seen in previous studies (Blank et al., 2002), indicating that it is selectively responsive during the production of actions that make a sound. Inferior parietal regions may underlie somatosensory guidance of articulatory movement as IPL is known to be active during enforced somatosensory perturbation (Golfinopoulos et al., 2011) and is central to adaptation to sensory changes (Shum et al., 2011). Inferior parietal regions may come into play when in the presence of predicable errors (Shum et al., 2011) or when novel motor output is considered.

## Conclusions

These data have implications for how we understand auditory processing during speech production. Humans frequently vocalize in situations where others are also simultaneously vocalizing and have to be able to produce and perceive speech within a complex auditory scene. Despite the fact that speech production is affected when there are other people speaking, most studies looking at the cocktail party effect (Cherry, 1953) focus on how speech is perceived and not on how speech is produced (Cooke and Lu, 2010). These data provide some insight into how auditory regions respond during articulation and how vocalization production systems respond during concurrent auditory input. Our findings present novel advances and challenges to extant anatomical and computational models of speech production.

## Figures and Tables

**Table 1:** The coordinates from statistical parametric maps derived from the main comparisons (*t*-tests) of interest are listed in Table 1, along with the corresponding coordinates, cluster sizes and z scores. Foci of maximal activation were localized using cytoarchitechtonic and probabilistic atlases available within SPM5 (Eickhoff et al., 2005). Coordinates are given in MNI space. Numbers of voxels are listed for main peaks only, not subpeaks.

Anatomy	Hemisphere	Coordinates (x y z)	Voxels (k)	z-Score
*ReadAloud_(ownvoice)_ > MouthSilently_(othervoice)_*				
	L	− 6 − 4 4	4932	5.08
Pallidum	R	18 − 1 − 5		4.94
Supplementary motor area BA6	L	− 3 11 64		4.89
Superior temporal gyrus	R	48 − 31 1	118	4.43
	R	39 − 46 7		2.78
Middle occipital gyrus BA 18	L	− 30 − 85 7	1920	4.27
	R	36 − 70 16		4.06
Calcarine gyrus		0 − 76 16		3.99
	L	− 42 − 43 1	27	3.47
Middle temporal gyrus	L	− 51 − 43 7		3.4
Superior medial gyrus	L	− 6 56 16	73	3.39
Superior medial gyrus	L	− 3 59 34		3.22
	L	− 27 − 37 13	24	2.9
	L	− 21 − 31 22		2.84

*MouthSilently_(othervoice)_ > ReadAloud_(ownvoice)_*				
Supramarginal gyrus	R	66 − 37 40	524	4.14
Angular gyrus	R	48 − 52 37		4.09
Superior parietal lobe	R	54 − 31 58		3.73
	L	− 63 − 40 43	264	3.83
	L	− 57 − 34 55		3.67
	L	− 60 − 58 31		3.38
Superior temporal gyrus				
(OP4 10%, TE1 10%)	L	− 60 − 13 4	25	3.06

*ReadSilently_(othervoice)_ > MouthSilently_(othervoice)_*				
Superior temporal gyrus	R	63 − 10 − 8	1372	5.22
Superior temporal gyrus	R	57 − 4 − 8		5.13
Precuneus	R	3 − 61 22		4.42
Superior temporal gyrus	L	− 45 − 16 1	392	4.78
Rolandic operculum OP2	L	− 36 − 25 16		4.21
Superior temporal gyrus	L	− 54 − 22 4		4.13
Superior temporal gyrus	R	24 38 52	74	4.47
Superior temporal gyrus	R	15 35 61		3.91
Superior medial gyrus	R	9 59 40		2.72
	R	3 2 − 8	334	4.4
Superior frontal gyrus	L	− 12 65 25		4.26
Caudate Nucleus	R	6 11 − 2		3.99
	L	− 39 − 79 43	326	4.39
Angular gyrus	L	− 48 − 70 37		4.03
Angular gyrus	L	− 39 − 61 28		4.03
Angular gyrus	R	51 − 64 25	313	4.26
Middle occipita gyrus	R	33 − 67 28		3.5
Angular gyrus	R	45 − 70 46		3.48
Inferior frontal gyrus (pars opercularis) BA 45 30%	L	− 54 23 34	89	4.2
Inferior frontal gyrus (pars triangularis BA 45 60%	L	− 51 20 19		3.29
Inferior frontal gyrus (pars triangularis BA 44 20%	L	− 42 17 31		3.19
Middle frontal gyrus	L	− 27 32 52	47	3.72
Superior frontal gyrus	L	− 18 32 58		3.51
Inferior frontal gyrus (pars triangularis) BA 44 50%	R	54 32 10	118	3.24
Inferior frontal gyrus (pars opercularis)	R	39 14 28		3.22
Inferior frontal gyrus (pars triangularis BA 45 10%	R	42 26 22		3.01
Paracentral lobule	L	− 3 − 25 61	34	3.19
Supplementary area BA 6	R	3 − 19 55		3.03

*ReadSilently_(othervoice)_ > ReadAloud_(ownvoice)_*				
Angular gyrus	R	54 − 64 25	960	6.37
Superior temporal gyrus	R	63 − 7 − 2		5.66
Superior temporal gyrus	R	66 − 19 7		4.38
	L	− 45 − 76 40	482	4.89
Angular gyrus	L	− 54 − 67 31		4.24
Angular gyrus	L	− 42 − 64 25		4.04
Superior temporal gyrus	L	− 45 − 16 1	281	4.86
	L	− 66		4.34
	L	− 84		4.31
Precuneus	L	− 6 − 52 19	427	4.14

*Null conjunction: [ReadSilently_(othervoice)_ > ReadAloud_(ownvoice)_] and [ReadSilently_(othervoice)_ > MouthSilently_(othervoice)_]*				
Superior temporal gyrus	R	63 − 10 − 5	259	5.09
Superior temporal gyrus	R	51 − 10 − 5		4.21
Superior temporal gyrus	R	72 − 28 4		4.11
Superior temporal gyrus	L	− 45 − 16 1	137	4.78
Superior temporal gyrus	L	− 54 − 22 4		4.12
Middle temporal gyrus	L	− 66 − 19 1		3.51
Angular gyrus	L	− 42 − 76 40	113	4.3
Angular gyrus	L	− 48 − 70 37		4.03
Angular gyrus	L	− 42 − 64 28		3.87
Angular gyrus	R	51 − 64 25	62	4.26
Precuneus	L	− 6 − 52 19	133	4.14
Precuneus	R	3 − 58 25		3.99
Angular gyrus	R	45 − 70 46	6	3.48
